# A Review of the Opioid Analgesic Benzhydrocodone-Acetaminophen

**DOI:** 10.7759/cureus.2844

**Published:** 2018-06-20

**Authors:** Ahmaya A Mustafa, Robin Rajan, Jennifer D Suarez, Saeed K Alzghari

**Affiliations:** 1 School of Pharmacy, Texas Tech University Health Sciences Center, Amarillo, USA; 2 School of Pharmacy, Texas Tech University Health Sciences, Abilene, USA; 3 School of Pharmacy, University of North Texas Health Science Center, Fort Worth, USA; 4 Genomics, Gulfstream Diagnostics, Dallas, USA

**Keywords:** benzhydrocodone, hydrocodone, opioids, abuse, misuse, prodrug, analgesics, opioid crisis

## Abstract

There is an undeniable opioid crisis in the United States that has caused significant negative consequences including many lives lost due to opioid overdoses. Currently, researchers are searching for alternatives for pain management as well as developing abuse-deterrent agents. In February 2018, the Food and Drug Administration (FDA) approved benzhydrocodone and acetaminophen (Apadaz™) for the short-term (no more than 14 days) management of acute pain severe enough to require an opioid analgesic and where alternative treatments are inadequate. This article looks further into this oral opioid prodrug and assesses its clinical pharmacology, pharmacokinetics, clinical trials and safety considerations that led to approval. Even though this prodrug provides a novel approach to analgesia, it was not classified as an abuse-deterrent agent and therefore still has the potential for abuse and misuse. This new agent potentially runs a higher risk of augmenting the opioid crisis rather than curtailing it. Innovative approaches to discover opioid alternatives are warranted.

## Introduction and background

**Figure 1:**
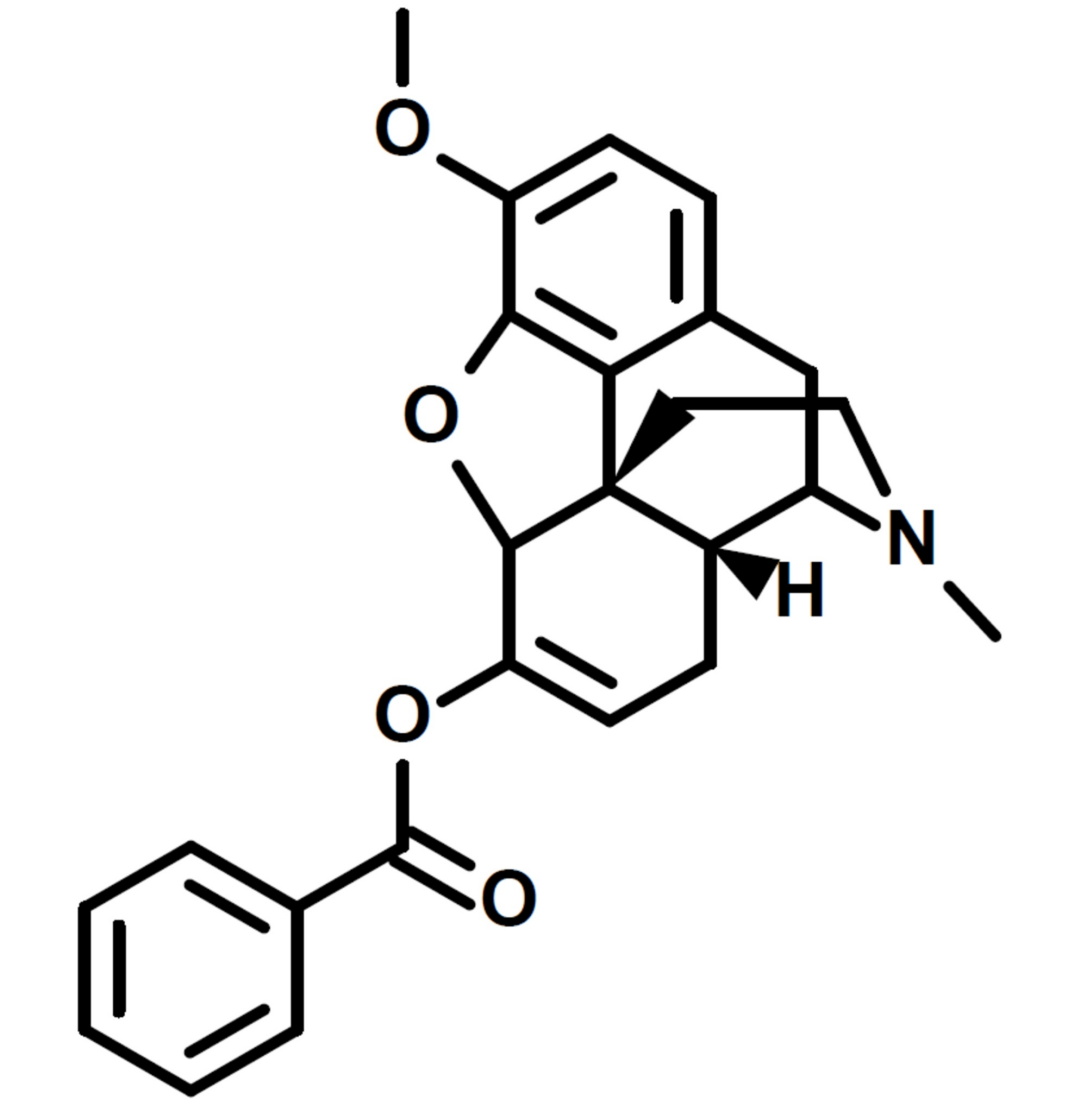
Structure of benzhydrocodone.

America is currently in an opioid crisis. In 2016, the incidence of opioid overdoses increased 500% compared to 1999. More than 115 American lives are lost due to opioid overdoses every day [1]. Annually, this results in more than 41,957 dead Americans due to opioid toxicity. In addition to lives lost, the Centers for Disease Control and Prevention (CDC) has also recognized an economic burden of $78.5 billion annually [2]. Collaboration across the disciplines is needed and it is vital that all healthcare providers are aware of this crisis. Emerging consequences from this issue, such as neonatal abstinence syndrome and the spread of infectious diseases through the use of injectable drugs, are increasingly becoming a healthcare issue [2]. The National Institutes of Health (NIH) has focused on preventing opioid misuse, treating opioid use disorder, and optimizing pain management. The NIH promotes drug dependence treatment, whether it be with the aid of medication or technology, and advocates improving overdose prevention and reversal to save lives [1]. In the midst of this crisis and the efforts to fight it, another opioid has been introduced to the market. On February 23, 2018, the Food and Drug Administration (FDA) approved Apadaz™, benzhydrocodone and acetaminophen (APAP), for the short-term (less than 14 days) management of acute pain severe enough to require an opioid analgesic and where alternative treatments are inadequate. It is comprised of benzhydrocodone (Figure 1), a synthetic opioid, and APAP, a non-opioid analgesic. The manufacturer of benzhydrocodone/APAP, KemPharm® (Coralville, IA, USA), believes the unique prodrug properties of benzhydrocodone/APAP will lead to commercial success, despite the plethora of opioids and other analgesics in the market [3]. Despite the manufacturer’s effort to create a diversion from the opioid crisis, benzhydrocodone/APAP is not classified as an abuse-deterrent drug. The potential benefits of benzhydrocodone/APAP are due to its prodrug approach where absorption is best achieved with oral administration as opposed to non-oral administration routes such as insufflation or injection [4]. Herein, we provide an overview of benzhydrocodone/APAP as well as its role in clinical practice.

## Review

Clinical pharmacology [5]

Benzhydrocodone is a prodrug of hydrocodone.

Hydrocodone is a full agonist of the opioid receptors with a higher affinity for the mu-opioid receptor. Upon binding, hydrocodone produces an analgesic effect with no ceiling. The exact mechanism of analgesia is unknown.

Acetaminophen is a non-opioid, non-salicylate analgesic. The specific mechanism of analgesia has not been determined.

Effects on the Central Nervous System

Hydrocodone causes respiratory depression and miosis.

Effects on the Gastrointestinal Tract

Hydrocodone decreases gastrointestinal (GI) motility by increasing smooth muscle tone and decreasing propulsive contractions, which may result in constipation.

Effects on the Cardiovascular System

Hydrocodone causes dilation of peripheral blood vessels, which can cause hypotension and syncope. Vasodilation coupled with histamine release can result in pruritus, sweating and flushing.

Effects on the Endocrine System

Hydrocodone, like other opioids, stimulates the secretion of prolactin, growth hormone (GH), insulin and glucagon. Conversely, hydrocodone inhibits adrenocorticotropic hormone (ACTH), cortisol and luteinizing hormone (LH) secretion. Chronic hydrocodone use can lead to androgen deficiency, which may result in low libido, impotence, erectile dysfunction, amenorrhea or infertility.

Effects on the Immune System

Hydrocodone use can suppress the immune system.

Pharmacokinetics [5]

Absorption

Bioequivalence studies have shown that benzhydrocodone/APAP is bioequivalent to other immediate-release hydrocodone combination products such as 7.5 mg hydrocodone/200 mg ibuprofen (Vicoprofen®) and 7.5 mg hydrocodone/325 mg acetaminophen (Norco®).

Metabolism

Benzhydrocodone is metabolized to hydrocodone by intestinal enzymes.

Hydrocodone can undergo O-demethylation via CYP2D6, N-demethylation via CYP3A4 and 6-keto reduction. O-methylation of hydrocodone produces hydromorphone, a potent opioid.

Acetaminophen is metabolized in the liver via glucuronide conjugation, sulfate conjugation or oxidation. The CYP450 dependent (CYP1A2, CYP2E1, and CYP3A4) oxidation pathway produces a reactive metabolite that conjugates with glutathione. The glutathione conjugate is then metabolized to cysteine and mercapturic acid conjugates.

Elimination

Hydrocodone is mainly excreted in the urine. The average half-life of hydrocodone is 4.5 hours. Acetaminophen metabolites are also eliminated in the urine. The average half-life of acetaminophen is 2 to 3 hours in adults.

Trials leading to FDA approval

Clinical Trials

A critical double-blinded, randomized crossover trial in 51 healthy adults, who identified as non-dependent recreational opioid abusers, demonstrated the pharmacokinetics and abuse potential of intranasal (IN) benzhydrocodone [6].

Patients were randomized into a double-blind, cross-over treatment sequence to receive equivalent doses of hydrocodone in the form of 13.34 mg of IN benzhydrocodone and 15 mg of IN hydrocodone bitartrate, separated by a 72-hour washout period. Blood samples were collected and peak plasma hydrocodone levels as well as total hydrocodone exposures were measured. Drug liking scores, safety and nasal assessments, along with ease of insufflation were assessed throughout each dosing interval [6].

The cohort treated with IN benzhydrocodone showed a significantly lower peak plasma concentration (C_max_ = 36% lower, p < 0.0001), significant delays in reaching peak effects (median of 1.75 hours vs 0.5 hours, respectively, p < 0.0001) and lower total hydrocodone exposures AUC_last_ (20.3% lower) and AUC_inf_ (19.5% lower), compared to hydrocodone bitartrate [6]. Additionally, IN benzhydrocodone showed a 47% lower abuse quotient (17% vs 31.9%), significantly lower drug liking scores (E_max_ = 67.4 vs 73.2, SD = 13.3, 95% CI = -1.9 to -9.6, p = 0.004) and difficulty to insufflate (78.7, SD = 20.0 vs 65.6, SD = 26.3, 95% CI = 19.4 to 5.9, p = 0.0004), when compared with IN hydrocodone bitartrate, demonstrating the abuse-deterrent characteristics of benzhydrocodone [6].

Another key randomized, double-blind, double dummy, two-part study, evaluated the bioavailability, safety and IN abuse potential of benzhydrocodone/APAP when compared to hydrocodone bitartrate/APAP in 42 healthy adults, with a history of non-dependent, recreational IN opioid abuse [4].

Patients from a dose selection phase (Part A) were randomized in the main study (Part B) to receive five doses of IN and oral benzhydrocodone/APAP (13.4 mg/650 mg), hydrocodone bitartrate/APAP (15 mg/650 mg) and placebo in one of 10 crossover sequences, separated by a minimum of a 96-hour washout period between treatments [4]. Assessments included the drug’s primary pharmacodynamics endpoint, drug liking peak effect (E_max_), assessed on a visual analog scale (VAS), as well as time to peak effect (TE_max_) and area under the drug liking effect curve (AUE) at five different time intervals. Safety and nasal effects assessments as well as ease of insufflation were also determined in each dosing period [4].

There were no significant differences in the primary pharmacodynamic endpoints of peak E_max_ and TE_max_ at different time intervals, but there was a significantly lower systemic hydrocodone exposure at early time intervals by approximately 50% (AUC_0-0.5 hrs_), 29% (AUC_0-1 hrs_), and 15% (AUC_0-2 hrs_) for IN benzhydrocodone/APAP compared to IN hydrocodone/APAP (p < 0.01, 90% CI for all comparisons) [4]. Unlike hydrocodone/APAP, the prodrug benzhydrocodone/APAP showed similar bioavailability for oral and intranasal routes of administration with an approximate 11% decrease in the intranasal C_max_ (mean = 34.7 ng/ml vs 39.1 ng/ml, p = 0.0027). Additionally, subjects receiving IN benzhydrocodone/APAP showed significantly greater nasal irritant effects across several different nasal effect measures and higher VAS scores for ease of insufflation (i.e., more difficult) when compared with hydrocodone/APAP (benzhydrocodone/APAP mean = 57.0, SD = 35.7, p = 0.01; hydrocodone/APAP mean = 43.4, SD = 32.5). All these factors yielded a 33% lower abuse quotient, providing less incentive for IN abuse of benzhydrocodone/APAP compared with hydrocodone/APAP [4].

Bioequivalence Studies

Two studies conducted by KemPharm®, demonstrated the relative bioequivalence of benzhydrocodone/APAP using two NDA-listed drug combinations. Vicoprofen® compared the hydrocodone bitartrate component (study 105) and Ultracet® (37.5 mg tramadol/325 mg APAP) compared the APAP component (study 106) [7].

Both studies were open-labeled, randomized, single dose, 2-treatment, 2-period, and 2-sequence crossover relative bioequivalence studies. Results from 28 subjects in study 105 and 27 subjects in study 106, demonstrated equivalence in exposure to hydrocodone, hydromorphone (active metabolite of hydrocodone), and APAP as measured by C_max_, AUC_0-t_, and AUC_inf_ (equivalent least squares mean ratio within 80%-125%, 95% CI) after oral administration of benzhydrocodone/APAP and the relative reference product [7].

Another set of relative bioequivalent studies were conducted comparing benzhydrocodone/APAP to hydrocodone/APAP in fed and fasting states to assay the effect of food on oral bioavailability and pharmacokinetics of benzhydrocodone/APAP [7].

Study 102 (oral dose of Norco® tablet, 7.5 mg hydrocodone/325 mg APAP, in 24 healthy subjects) looked at the effects of the agent in the fasting state [7]. Benzhydrocodone/APAP showed bioequivalent pharmacokinetics parameters for hydrocodone as discussed above within the acceptable range (80%-125%, 95% CI).

Benzhydrocodone/APAP also showed similar pharmacokinetics bioequivalent results for APAP, within a slightly lower range (79.8%); however, consideration of the results from the Ultracet® study deemed the range acceptable. The data for hydromorphone could not be reliably estimated due to the lack of a log-linear decay for many of the hydromorphone datasets, and only four subjects had a value for both treatments.

Study 104 (oral dose of benzhydrocodone/APAP 6.67 mg/325 mg tablet vs Norco®, in 38 healthy subjects) looked at the effects of the agent in the fed state [7]. Comparable median times and identical ranges were observed to maximum exposure (T_max_) for hydrocodone and APAP. When administered with food, the data demonstrated a small decrease in the exposure rate (least squares mean ratio of C_max_ = 78.4%) and similar overall exposure to hydrocodone (AUC_last_ and AUC_inf_) when compared with Norco®, suggesting no safety concern or impact on the efficacy of benzhydrocodone/APAP in the fed state.

For benzhydrocodone/APAP in the fed and fasted condition, overall extent of exposure (AUClast and AUCinf) was equivalent to hydrocodone and APAP, but slightly lower peak exposure (C_max_: least squares mean ratio = 85.3%) was observed. Overall, these results suggest that benzhydrocodone/APAP can be administered regardless of food [7].

Boxed warnings [5]

Addiction, Abuse and Misuse

Benzhydrocodone/APAP has the potential for addiction as well as abuse and misuse, which can result in overdose and death.

Respiratory Depression

Serious, life-threatening, or fatal respiratory depression can occur.

Accidental Ingestion

Accidental ingestion can cause a fatal overdose of hydrocodone.

Neonatal Opioid Withdrawal Syndrome

Hydrocodone can cause neonatal opioid withdrawal syndrome when taken during pregnancy.

Hepatotoxicity

Benzhydrocodone/APAP contains acetaminophen, which can cause acute liver failure at high doses (>4000 mg a day in healthy adults).

Cytochrome P450 3A4 Interaction

Benzhydrocodone/APAP use with CYP3A4 inhibitors can cause a fatal overdose of hydrocodone.

*Risks from Concomitant Use with Benzodiazepines or Other Central Nervous System* *(CNS) Depressants*

Benzhydrocodone/APAP use with benzodiazepines or other CNS depressants can cause sedation, respiratory depression, coma or death.

Drug abuse and dependence [5]

Benzhydrocodone is a schedule II-controlled substance. Like other opioids, benzhydrocodone has the potential to be abused.

Chronic benzhydrocodone use can lead to the development of tolerance and physical dependence. Withdrawal symptoms can occur from abrupt discontinuation or rapid tapering of benzhydrocodone.

Adverse events [5]

The safety of benzhydrocodone/APAP was evaluated in a total of 200 healthy adults in six phase 1 studies. The subjects received at least one oral dose of benzhydrocodone/APAP. The most common adverse events in these studies were nausea (21.5%), somnolence (18.5%), vomiting (13.0%), constipation (12.0%), pruritus (11.5%), dizziness (7.5%) and headache (6.0%).

Drug-drug interactions [5]

Serotonergic Drugs

Benzhydrocodone/APAP use with serotonergic drugs can cause serotonin syndrome.

Mixed Agonist/Antagonist and Partial Agonist Opioid Analgesics

Mixed agonist/antagonist and partial agonist opioids may reduce the analgesic effect of benzhydrocodone/APAP or cause withdrawal symptoms.

Monoamine Oxidase Inhibitors (MAOIs)

MAOIs can increase the effects of benzhydrocodone/APAP.

Contraindications [5]

Benzhydrocodone/APAP is contraindicated in patients with significant respiratory depression, bronchial asthma in an unmonitored setting, known or suspected gastrointestinal obstructions (including paralytic ileus). It is also contraindicated in patients that have experienced hypersensitivity to hydrocodone, acetaminophen or any other component in the formulation.

Use in specific populations [5]

Pregnancy

Hydrocodone can cause neonatal opioid withdrawal syndrome when taken during pregnancy.

Reproductive and developmental studies of acetaminophen in rats and mice have shown fetotoxicity, necrosis of the liver and kidney in the pregnant rat and the fetus, retarded growth and decreased reproductive capacity of the offspring.

Lactation

Hydrocodone and acetaminophen are present in breast milk.

Females and Males of Reproductive Potential

Chronic opioid use can cause infertility.

Pediatric Use

Safety and efficacy has not been established in patients under the age of 18.

Geriatric Use

Patients over the age of 65 may have heightened sensitivity to hydrocodone. Benzhydrocodone/APAP is mainly excreted through the kidneys; therefore, geriatric patients with impaired renal function may experience more adverse reactions.

Renal Impairment

The effect of renal impairment on the pharmacokinetics of benzhydrocodone/APAP has not been determined.

Hepatic Impairment

The effect of hepatic impairment on the pharmacokinetics of benzhydrocodone/APAP has not been determined.

Dosage and administration [5]

Each benzhydrocodone/APAP immediate release tablet contains 6.12 mg of benzhydrocodone (equivalent to 6.67 mg of benzhydrocodone hydrochloride) and 325 mg of acetaminophen. The tablets are white, capsule-shaped and are debossed with “KP201” on one side.

Dosing should start at one to two tablets every four to six hours, as needed for pain. The dosage should not exceed 12 tablets in a 24-hour period. The total dosage of benzhydrocodone/APAP and any other acetaminophen containing products should not exceed 4000 mg of acetaminophen in a 24-hour period.

Discussion

A prodrug opioid is a novel approach to analgesia; however, the approval of benzhydrocodone/APAP is not a revolutionary treatment option for pain. Furthermore, benzhydrocodone/APAP may not reduce the prevalence of opioid abuse. A prodrug allows for the enhancement of the drug by controlling the solubility, lipophilicity, bioavailability, half-life, extended-release profile, interpatient variability, and targeted tissue/organ delivery [8]. However, despite these advantages, prodrugs are only a few steps away from the parent drug. A prodrug does not mean a drug is less potent, as the design of a prodrug varies and could be very complex. In other words, a prodrug could potentially allow the provider better control regarding toxicity to opioids. One may view benzhydrocodone/APAP as a safer option due to its prodrug properties, but one could argue otherwise. The most common route for the abuse of hydrocodone combination products is oral administration (90.3%) [9].

Benzhydrocodone/APAP has reduced bioavailability in non-oral routes of administration, but it is still susceptible to oral abuse [3]. Furthermore, benzhydrocodone/APAP did not receive classification as an abuse-deterrent opioid, which demonstrates benzhydrocodone/APAP does not have less potential for abuse. Therefore, a decrease in opioid abuse is not likely to occur due to benzhydrocodone/APAP approval. In fact, benzhydrocodone/APAP has the potential to contribute to the opioid crisis by becoming an additional drug option for individuals to abuse. The market already contains established opioids with proven efficacy and prescriber familiarity such as morphine, fentanyl, oxycodone and hydrocodone. The main drawbacks of these drugs are physical dependence and abuse potential. During the period between 2010 and 2015, the rates of death involving heroin and synthetic opioids, other than methadone, increased across all demographic groups, regions, and in numerous states [1]. From 2014 to 2015, deaths from synthetic opioids increased by 72% [10]. On April 4, 2018, the NIH launched the HEAL (Helping to End Addiction Long-term) Initiative. Congress has allotted $1.1 billion in research funding for this initiative intended to expedite scientific solutions for this public health crisis [11]. The focal point of this action is to reduce the prescription of opioids, to advance effective non-opioid therapies for pain, and give patients autonomy in treatment selection for their opioid addiction. Innovative steps toward fighting this opioid crisis are creating an agent with new targets for the treatment of chronic pain and discovering objective biomarkers that predict which individuals will respond to a treatment [11].

From 1998 to 2012, the United States production of hydrocodone increased more than four-fold [12]. In 2010, the United Nations estimated a global hydrocodone production of 36.3 tons; the United States reportedly consumed 99% [12]. Hydrocodone is prescribed and dispensed more than all other medicinal opioids combined [12]. In 2015, nearly 300 million narcotic pain medication prescriptions were dispensed around the world; Americans constituted 80% (240 million) [13]. It is critical that we consider all aspects and consequences of prescribing and dispensing an opioid. Benzhydrocodone/APAP is a novel prodrug opioid. However, it was only approved to treat short-term acute pain and it has not been confirmed as an abuse deterrent agent. Like other opioids, benzhydrocodone/APAP presents a potential for addiction, as well as abuse and misuse.

The FDA has supported the development of novel analgesic and abuse-deterrent opioid formulations. Their vision promotes the creation of pain management drug classes, the study of novel analgesic compounds that target different steps along the inflammation cascade and innovative technologies [14]. Creative approaches to develop alternatives to opioids need to be sought out in order to treat chronic pain as well as curtail the current opioid epidemic. Adding a prodrug of hydrocodone to the pool of pain management medications is highly unlikely to divert the opioid crisis. In fact, this new agent may potentiate the problem. The indication itself, “short-term acute pain,” is likely to attract many prescribers and patients. It is vital to consider all of the effects from this agent before prescribing especially within the larger context of the current opioid crisis.

## Conclusions

The recent approval of benzhydrocodone/APAP adds to the list of opioids available on the market. However, not receiving an abuse-deterrent classification from the FDA limits its potential in that regard. Time will determine the value of benzhydrocodone/APAP to clinicians and, ultimately, patients with respect to pain management as well as its risk for abuse. Further research to find opioid alternatives is warranted.
